# Supplementary material for: The DNA adenine methylase of Salmonella Enteritidis promotes their intracellular replication by inhibiting arachidonic acid metabolism pathway in macrophages
Source: Front Microbiol. 2023 Mar 2;14:1080851. doi: 10.3389/fmicb.2023.1080851 (PMC10018194; doi:10.3389/fmicb.2023.1080851)
Supplement: Supplementary file 2 [file Data_Sheet_2.zip › S1 Appendix. Non-targeted metabolomics raw data/4.MetDiffAnalysis/C50336_Ddam.vs.control/C50336_Ddam.vs.control_pos_zscore.pdf]

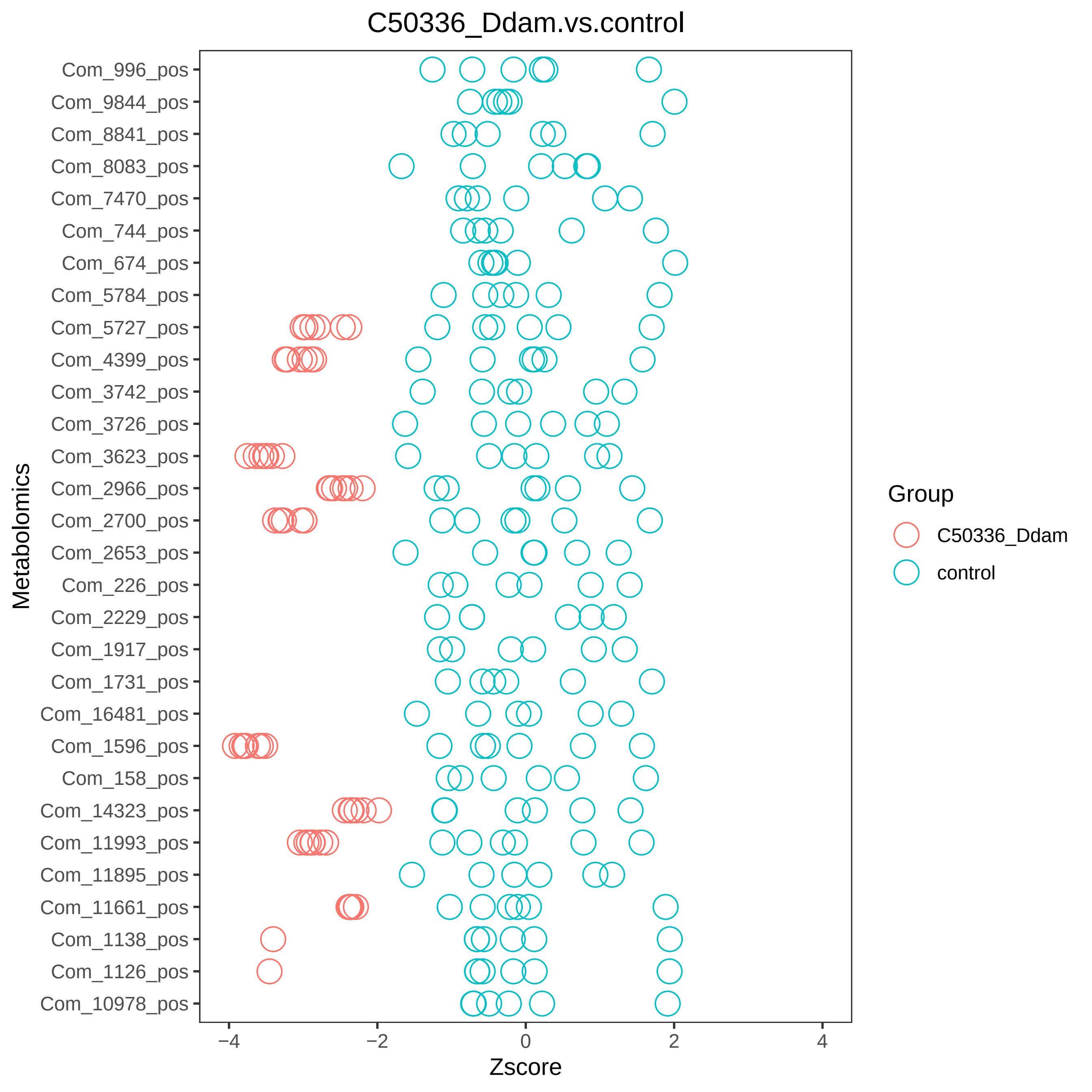

C50336_Ddam.vs.control
Com_996_pos
Com_9844_pos
Com_8841_pos
Com_8083_pos
Com_7470_pos
Com_744_pos
Com_674_pos
Com_5784_pos
Com_5727_pos
Com_4399_pos
Com_3742_pos
Com_3726_pos
Com_3623_pos
Com_2966_pos
Com_2700_pos
Com_2653_pos
Com_226_pos
Com_2229_pos
Com_1917_pos
Com_1731_pos
Com_16481_pos
Com_1596_pos
Com_158_pos
Com_14323_pos
Com_11993_pos
Com_11895_pos
Com_11661_pos
Com_1138_pos
Com_1126_pos
Com_10978_pos
Metabolomics
−4
−2
0
2
4
Zscore
Group
C50336_Ddam
control
